# Comparative Study of *Callistemon citrinus* (Bottlebrush) and *Punica granatum* (Pomegranate) Extracts for Sustainable Synthesis of Silver Nanoparticles and Their Oral Antimicrobial Efficacy

**DOI:** 10.3390/nano14110974

**Published:** 2024-06-04

**Authors:** Enas Ismail, Abubaker Mohamed, Amir Elzwawy, Ernest Maboza, Mokhotjwa Simon Dhlamini, Razia Z. Adam

**Affiliations:** 1 Department of Prosthodontics, Faculty of Dentistry, University of the Western Cape, Cape Town 7505, South Africa; 2 Physics Department, Faculty of Science (Girl’s Branch), Al Azhar University, Nasr City 11884, Cairo, Egypt; 3 Ceramics Department, Advanced Materials Technology and Mineral Resources Research Institute, National Research Centre (NRC), 33 El Bohouth St., Dokki, Giza 12622, Egypt; 4 Oral and Dental Research Laboratory, Faculty of Dentistry, University of the Western Cape, Cape Town 7505, South Africa; 5 Department of Physics, University of South Africa, Private Bag X6, Florida 1710, South Africa

**Keywords:** silver NPs, green synthesis, *Punica granatum* (pomegranate) peel extract, *Callistemon citrinus* (bottlebrush) flower extract, antimicrobial activity

## Abstract

A comparative study was applied to investigate the potential of *Callistemon citrinus* (bottlebrush) flower extract (BBE) and *Punica granatum* (pomegranate) peel extracts (PPE) for the sustainable synthesis of the silver nanoparticles, Ag-BBE and Ag-PPE, respectively. The synthesis process of Ag NPs using the selected extracts was applied under optimized conditions. Hence, the effect of the selected plant’s type on the different characteristics of the synthesized green Ag NPs was investigated. The UV-Vis spectroscopy revealed the presence of the characteristic silver peaks at 419 and 433 nm of the Ag-BBE and Ag-PPE, respectively. The XRD spectra reported the fcc phase formation of Ag NPs. The TEM results highlighted the morphological features of the synthesized Ag NPs. with a size range of 20–70 nm, and with 10–30 nm for Ag-BBE and Ag-PPE, correspondingly. The Raman spectra revealed characteristic silver bands in the Ag-PPE and reflected some bands related to the natural extract in the Ag-BBE sample. The antimicrobial activity and statistical analysis investigation were conducted against four selected oral pathogens (*Staphylococcus aureus* (*SA*), *Candida albicans* (*CA*), *Staphylococcus epidermidis* (*S. epi*), and *Enterococcus faecalis* (*EF*)). Both tested extracts, BBE, and PPE, revealed potential effectivity as reducing and capping agents for Ag NP green synthesis. However, the synthesized NPs demonstrated different features, depending on the used extract, reflecting the influence of the plant’s biomolecules on the nanoparticles’ properties.

## 1. Introduction

Oral diseases are regarded as a major health burden, impacting a large group of people belonging to different age groups around the world. According to the World Health Organization (WHO), around 3.5 billion people around the world are affected [1]. They are predominantly caused by compromised teeth with dental caries, which can involve the pulp tissues and spread to the surrounding tissues. Furthermore, soft tissue involvement comprises the gingival and periodontal tissues, as well as the oral mucosa. These oral disorders are considered non-communicable but cause discomfort, suffering, and, in severe cases, tooth loss, which can afflict people of any age [2,3]. Oral plaque is composed of polymicrobial species (bacteria and fungi) that can resist the host’s immune mechanisms and lead to the occurrence of oral diseases [4]. *Candida albicans* is one of the major species forming dental plaque. The nature of a candidal infection is an opportunistic infection, where the body’s immunity is compromised. It can induce oral candidiasis and can cause root canal treatment failure [5,6]. Other species such as *Staphylococcus aureus* are accountable for many oral diseases, such as dental caries, oral mucositis, peri-implantitis, periodontitis, and endodontic infections [7,8,9]. Furthermore, *Enterococcus faecalis* is an aerobic Gram-positive coccus that is found in the oral cavity, vagina, and gastrointestinal system. Furthermore, *E*. *faecalis* is responsible for secondary endodontic infections and the failure of root canal treatment [10].

The requirement for potent antimicrobial agents in dental materials is of paramount importance in combating the resistant microorganisms that cause oral disease. A novel substitute for treating oral diseases caused by pathogens is the use of green nanomaterials [11]. It has been reported that nanoparticles show broad-spectrum antimicrobial activity against a wide range of microbes, including Gram-positive and Gram-negative bacteria, fungi, and viruses [12]. The noble metal nanoparticles (Ag, Au, Pd, Pt, Os, Ru, Rh, and Ir) with their distinctive physiochemical and biological properties have been exploited widely in materials science, medicine, biology, chemistry, and physics [13]. The antimicrobial activity of the synthesized nanoparticles depends on their physiochemical features, including their size, morphology, surface charge, zeta potential, and phase structure [14].

Among the noble metal nanoparticles, Ag nanoparticles exhibited excellent antimicrobial activity against oral bacterial and fungal pathogens [15,16,17]. Ag nanoparticles, when compared to Ag+, revealed enhanced antimicrobial potential [18,19]. Ag nanoparticles have a greatly toxic effect on microorganisms [18]. However, it is essential to consider the lower toxicity concentration of Ag nanoparticles in mammalian cells. The antimicrobial mechanism of Ag nanoparticles was extensively investigated but is not yet fully understood. Different prospective mechanisms of Ag nanoparticles against different bacterial species were reported by Pragati Rajendra More in 2023 [20]. More et al. reveal the action mode of Ag nanoparticles against different Gram-positive and -negative strains. Ag nanoparticles can accumulate at the outer membrane owing to electrostatic attraction, causing membrane denaturation and perforation. Ag nanoparticles can inhibit growth and metabolism within the bacterial cell [20]. They can attach to the cell membrane, distract respiration, and affect bacterial permeability [20]. Ag nanoparticles are capable of releasing Ag+ ions that disrupt the outer membrane and cytoplasmic membranes, followed by entering the cell, causing ribosomal denaturation, and interfering with the replication of the DNA. In a further mechanism, Ag nanoparticles release reactive oxygen species (ROS) that cause membrane disruption and interfere with DNA replication [21].

Based on the potential activity of Ag nanoparticles as an antimicrobial agent, different and distinct approaches have been employed for the synthesis of Ag nanoparticles, such as laser ablation [22], thermal treatment [23], wet chemical treatment [24], photochemical reduction [25], metal vapor decomposition [26], and sputtering [27]. The main disadvantages of using the physical and chemical methods are the chemical cost, the possible need for high-energy production, and the generation of products with toxicity levels that are not suitable for biomedical applications [28,29]. A bottom-up approach is an eco-friendly method where green synthesis is utilized. In this method, reduction and oxidation are the primary reactions for producing nanoparticles [30,31,32,33,34,35,36,37,38,39,40]. This environmentally friendly strategy employs less damaging substances to health by utilizing natural resources such as plants (leaf, bark, flower, fruit, and root extracts) and micro-organisms (bacteria, fungi, viruses, and algae) [31,33]. The foremost benefits of utilizing the green approach are reduced cost, availability, ease of manipulation, and biocompatibility [34]. Various plant extracts with distinct secondary metabolites, including *Achillea maritima* [35], *Coriandrum sativum* [33], *Symphyti radix* [36], *Lavandula angustifolia* Mill [37], *Avena fatua* L. [38], *Holigarna arnottiana* [39], *Pulicaria undulata* [40], and *Leucosidea sericea* [32] were recently employed as reducing and capping agents in the environmentally friendly synthesis of Ag nanoparticles.

The pomegranate tree is scientifically known as *Punica granatum*. The cultivars of pomegranate are distributed across the five continents and are mainly harvested in India, China, Iran, Afghanistan, and the Middle Eastern region [41,42]. Pomegranate peel is considered a waste product and comprises around 30–40% of the total fruit weight [43]. Pomegranate peel is rich in phenolic compounds, flavonoids, tannins, and ellagitannins (punicalagin and punicalin), which are used in NP synthesis [44,45]. Conversely, *Callistemon citrinus* (bottlebrush) is a plant in the Myrtaceae family that is commonly named red bottlebrush, crimson bottlebrush, and lemon bottlebrush [46]. The red bottlebrush is an ornamental plant found in Australia, Africa, America, and Asia [47,48]. *Callistemon citrinus* extract is rich in phytochemicals, including flavonoids, alkaloids, terpenoids, sterols, and tannins [49,50]. Pomegranate and bottlebrush extracts were successfully reported in the green synthesis of different stable metallic nanoparticles [51,52]. Moreover, *Callistemon citrinus* extract is utilized to green-synthesize Au nanoparticles, and the resulting antimicrobial effect against a wide range of bacteria revealed strong activity [53].

To override the expected drawbacks of physical and chemical methods to improve human health, green methods are proposed as a safe and environmentally friendly approach. Thus, in this study, Ag nanoparticles will be synthesized using the green biosynthesis approach. In dentistry, to combat dental diseases and infections caused by harmful pathogens, the antimicrobial capacities of dental materials must be improved significantly. Ag nanoparticles are postulated as strong antimicrobial agents that have the potential to boost biological properties when incorporated in dental materials. This study aims to green-synthesize Ag nanoparticles using two different natural extracts, *Punica granatum* (pomegranate) peel extract (PPE) and *Callistemon citrinus* (Bottlebrush) flower extract (BBE), under similar conditions. The study objectives are to reveal the effect of the selected extract on the different characteristics and features of the nanoparticles. Their antibacterial efficacy against oral pathogens will also be investigated. 

## 2. Materials and Methods

### 2.1. Extraction Process

**Figure 1:**
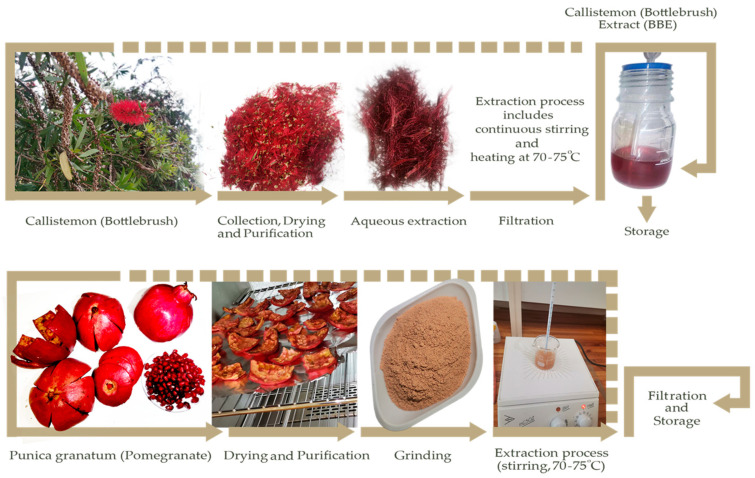
The extraction process of the two selected plants, *Callistemon* (Bottlebrush) and *Punica granatum* (pomegranate) peel extracts, respectively.

In the current research, *Callistemon citrinus* (bottlebrush) flower and *Punica granatum* (pomegranate) peel were used for the green synthesis of Ag NPs. The extract of *Callistemon citrinus* (bottlebrush) flower was named BBE, while the extract of *Punica granatum* (pomegranate) peel was named PPE. Hence, in the further synthesis of silver nanoparticles (Ag NPs), the samples were named Ag-BBE, and Ag-PPE NPs in respective of the used extract in the green synthesis process. Figure 1 represents the extraction process of the two selected plants, *Callistemon citrinus* (bottlebrush) and *Punica granatum* (pomegranate), separately. To prepare the *Callistemon citrinus* extract, the bottlebrush (BB) flowers were freshly collected in Cape Town, South Africa. The cleaning, purification, and gentle drying of the BB flowers are essential before the extraction process [54]. First, 50 mL of a green sustainable aqueous solvent (distilled H_2_O) was added to 1 g of the flowers and left under continuous stirring at 70–75 °C. The combined effect of stirring, temperature, and time is essential for the proper extraction of the naturally desired biomolecules and will have a vital role in nanoparticle synthesis. The change in the color of the extracts will be noticeable, changing from colorless to deep purple, which is a characteristic color for bottlebrush extract (BBE), as shown in Figure 1. The pH of the BBE was approximately 6. Further centrifugation at 5k rpm/10 min followed by Whatman filter paper filtration of the BBE was applied. The BBE was then stored at 4 °C for the further synthesis of Ag NPs. However, based on our experience, the BBE should NOT be stored for many days before its use in NP synthesis. Using a fresh extract is highly recommended to avoid contamination and the lower effectivity of the extract due to degradation or changes in the extract’s composition.

In the second step, the *Punica granatum* (pomegranate) peel extract (PPE) was applied as represented in Figure 1. The pomegranate fruits were collected from Cape Town, South Africa. Firstly, the fruit was cleaned using distilled H_2_O and peeled. The seeds were taken away, while the peels were rewashed and readied for drying. The drying process was conducted for 8 h at 70 °C before grinding using a ball mill grinder (Fritsch pulverisette, Heidelberg, Germany). The thickness of the peels notably affects the drying time. Furthermore, over-drying or increasing the drying temperature may cause severe damage and destruction to the peel composition. The grinding process converted the pale reddish peels into a light brownish powder, as displayed in Figure 1. Like the BBE, 1 g of the pomegranate powder was added to 50 mL of distilled water using a magnetic stirrer at 70–75 °C for the complete extraction process of *Punica granatum* (pomegranate) peel extract (PPE). The PPE was then filtered and stored in similar conditions as the BBE.

### 2.2. Plant-Mediated Synthesis of Silver Nanoparticles

The green synthesis process of Ag NPS using the two selected extracts, BBE and PPE, under the same conditions, to compare and evaluate the effect of the plant type on the synthesis process, and also to evaluate the effect made by the plants, under the same conditions, on the different characteristics and properties. This is to reveal the effect of the phytochemicals or biomolecules in each extract on the synthesis process. A stock solution of 10^−2^ M silver nitrate, acting as the main silver precursor, was prepared. To identify the optimum synthesis conditions for the silver nanoparticles (Ag NPs), different aspects were considered. This includes the concentration, the ratio between the silver precursor and the tested extracts and separately, the temperature, and the reaction duration. Noticeably, the effect of applying a different pH in the synthesis process of the NPs was not considered in the current report, to minimize adding any additional chemicals that may affect the final green product. However, the tested extracts revealed a similar pH of 6; hence, it is considered a constant parameter in the evaluation of optimal synthesis conditions using BBE and PPE. In the initial screening and optimization steps, the current report evaluated the effect of:

(a) Plant concentration: Different serial dilutions of PPE were applied.
(b) Volume ratio: three different ratios of extract: Ag precursor (1:1, 1:2, 2:1) were applied.
(c) Reaction time: Different time intervals, including 0 h, 0.5 h, 1 h, 1.5 h, 2 h, 2.5 h, 3 h, 3.5 h, and 4 h were applied. The selected optimal conditions for the green synthesis of Ag NPs using PPE were then applied for Ag NP synthesis using BBE. The green-synthesized silver nanoparticles, Ag-BBE and Ag-PPE, were then analyzed using different characterization techniques to analyze their different characteristics and properties. The optical properties of the synthesized samples were evaluated using UV-Vis spectroscopy (SPECTROstar Nano 2450, BMG LABTECH, Offenburg, Germany). Their crystallographic properties were studied using an x-ray diffractometer (Rigaku Smartlab, Tokyo, Japan) at 1.5406 Å, Cu Kα1 line). The morphological properties were studied through a high-resolution transmission electron microscope (HRTEM, JOEL-JEM 2100, Tokyo, Japan). Furthermore, the vibrational studies of the green-synthesized Ag NPs were examined using XploRA Plus Raman spectroscopy (Jobin–Yvon T64000, Palaiseau, France) via an Argon ion laser line of 514.5 nm. The antimicrobial properties were evaluated using an antimicrobial activity test and statistical analysis.

### 2.3. Antimicrobial Activity Test

The antimicrobial potential of the green-synthesized silver nanoparticles (Ag-BBE, and Ag-PPE) and the related aqueous extracts (BBE, and PPE) was examined against 4 microbes generally associated with the oral cavity. These microbes range from skin to gastrointestinal microflora with pathogenic potential. *Staphylococcus aureus* ATCC 6538 (*SA*), *Candida albicans* ATCC 10231 (*CA*), and *Enterococcus faecalis* ATCC 29212 (*EF*) were purchased from the American Type Culture Collection (Microbiologics, St Cloud, MN, USA), while the *Staphylococcus epidermidis* (*S. epi*) was donated by the Oral and Dental Research Laboratory, Faculty of Dentistry, University of the Western Cape, South Africa. All cultures were resuscitated in brain–heart infusion broth (BHI102453085; Sigma–Aldrich, Johannesburg, South Africa) and incubated at 37 °C for 24 h. Thereafter, the microbes were aseptically subcultured in brain–heart infusion agar (BHA 102177428; Sigma–Aldrich) and incubated for a further 24 h at 37 °C to achieve pure growth. Then, the fresh culture was adjusted to 0.5 McFarland standard (Mcf) in phosphate-buffered saline (PBS, P4417-100TAB, Sigma-Aldrich, Johannesburg, South Africa).

The Kirby–Bauer disc diffusion method was used to study the antibacterial activity of the different treatments. Sterilized filter-paper discs (8 mm/Ø, LASEC, Cape Town, South Africa) were used. The discs were aseptically infused stepwise with 100 μL of BBE, PPE, Ag-BBE NPs, and Ag-PPE NPs, plus chlorhexidine (CHX), separately. The concentration of the CHX used was 0.2% (H2962; Resmed Healthcare, San Diego, CA, USA). The infused discs were air-dried in a laminar flow cabinet under sterile conditions.

Then, 100 μL of each microbe, adjusted to 0.5 Mcf, was spread onto Mueller Hinton agar (MHA, 102467611; Sigma–Aldrich) with a sterile hockey stick. The dried discs were then placed on the cultured plates and the plates were thereafter incubated for 24 h at 37 °C. After incubation, the plates were observed for zones of inhibition (ZOI). A Vernier caliper was used to measure the ZOI of the different treatments (BBE, PPE, Ag-BBE, and Ag-PPE). The results were recorded in Excel for further statistical investigation. An analysis of variance (ANOVA) using GraphPad Prism version 6 was used at alpha *p* < 0.05. Furthermore, to elucidate the source of statistical significance or the lack thereof across the tested Ag NPs and their respective plant extracts, a Bonferroni post hoc test was used. This method was adapted from the work of Ismail et al. (2024), with minor adjustments [54].

## 3. Results and Discussion

### 3.1. Screening Step and Optimization

**Figure 2:**
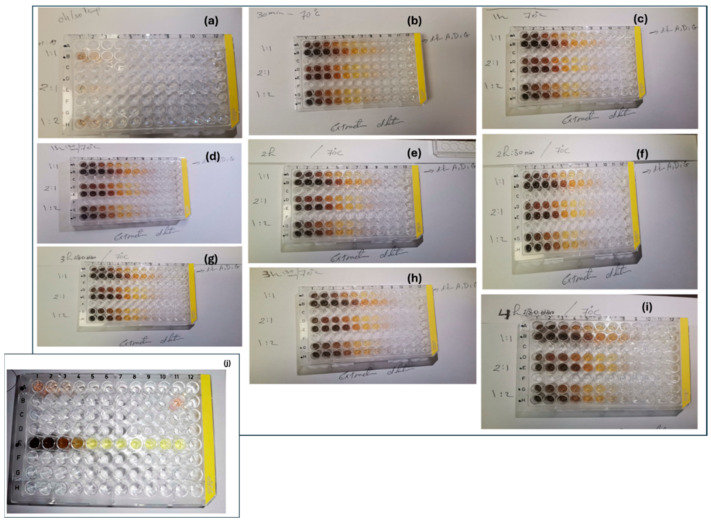
(**a**–**i**) The initial synthesis screening and optimization process of the green synthesis of Ag NPs using PPE and (**j**) BBE extracts.

An initial screening process was performed to evaluate the optimum synthesis ratios between the silver precursor solutions, the *Callistemon* (bottlebrush) extract (BBE), and the *Punica granatum* (pomegranate) peel natural extracts (PPE), separately. Three different volume ratios of 1:1; 1:2; and 2:1 between the plant extract (BBE and PPE, separately) and the silver precursor were applied to initially identify the optimal ratio for further processing (Figure 2). The effect of the PPE extract’s serial dilutions on the Ag NP green synthesis, in addition to the effect of temperature and reaction time, was investigated, as shown in Figure 2a–i. A total of 36 main synthesized samples was examined in each step. The initial results identified 70 °C as an optimal temperature and this has been considered a stable parameter in the currently listed investigation steps of the appropriate reaction time. The selected optimal temperature is in agreement with different published reports [55,56]. Different reaction periods were initially examined to identify the optimal time, as shown in Figure 2a–i. Figure 2a represents the instant and initial step of Ag NP formation. Figure 2b, Figure 2c, Figure 2d, Figure 2e, Figure 2f, Figure 2g, Figure 2h, and Figure 2i represent the tested ratios and extract concentrations at 70 °C for different time intervals (0.5 h, 1 h, 1.5 h, 2 h, 2.5 h, 3 h, 3.5 h, and 4 h), respectively. The initial indication of the formation of Ag NPs can be noted through the color changes of some samples. The change in the color can be attributed to the reduction process of the silver ions and the formation of Ag^0^. This is an initial indication of the effectiveness of the tested plant extracts as a reducing agent. It is reported that natural extracts contain phenols and flavonoids, etc., which can vitally reduce the silver ions to NPs [32]. Based on the initial screening of the effect of PPE on the synthesis process of Ag NPs, the optimized parameters of optimal temperature, ratio, and reaction duration were used for further Ag-BBE synthesis investigation (Figure 2j). This is to ensure a proper comparison between the potential effects of both extracts (PPE and BBE) on the synthesis process. However, a serial dilution of BBE was reapplied to ensure the proper optimal extract concentration. Further, UV-Vis spectroscopy was applied in each step for the listed samples to explore the absorption spectra and characteristics of surface plasmon resonance (SPR) of the synthesized Ag NPs [57,58]. The screening and optimization process, depending on the initial screening data of the UV spectroscopy process, revealed the effectivity of the green synthesis of Ag NPs using PPE at 70 °C for an hour (Figure 2d), while the ideal selected volume ratio is 1:2 of extract:silver ions. Noticeably, the increase in the reaction time (2–4 h) at 70 °C does not affect the SPR position of the Ag NPs, although it does enhance the absorption intensity. This may potentially be attributed to the enhancement of the Ag NPs’ crystallinity over time. The selected optimal conditions were employed for both extracts (BBE and PPE) to examine their potential ability in the synthesis of Ag NPs.

### 3.2. UV-Vis Spectroscopy Analysis (Optical Properties)

**Figure 3:**
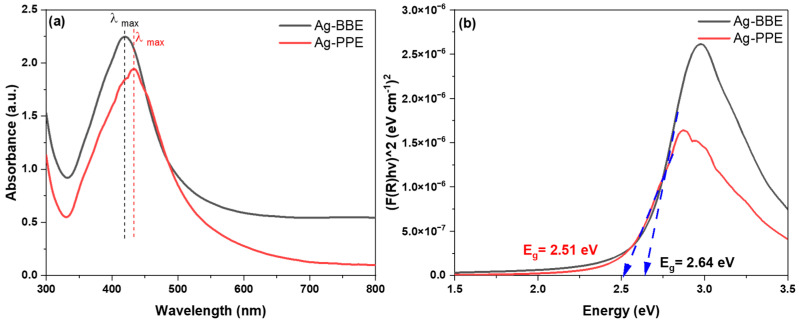
(**a**) The UV-Vis spectra of the silver nanoparticles prepared with both extracts and (**b**) Tauc plot for determination of the optical bandgap (*E_g_*) of the prepared silver nanoparticles, using the BBE and PPE extracts.

In this section of the results and discussion, we will reveal the basic specifications of the green-synthesized Ag NPs using BBE and PPE natural plant extracts. The formed nanoparticles are investigated through a set of conventional characterization devices. UV-Vis spectroscopy is a very beneficial and dependable method for the main characterization of produced nanoparticles, which also functions to monitor stability and the formation of nanoparticles beyond merely the color change indication [59,60,61]. The green-synthesized samples were examined using a UV–Vis spectrophotometer within the spectral range of 200 to 900 nm to obtain the absorbance spectra. The UV-Vis spectra shed light on the free surface electrons’ collective oscillations with incident light, known as surface plasmon resonance (SPR). The SPR reflects the collective oscillations of the conduction electrons of the explored structure [62,63]. The UV-Vis results are highly dependent and affected by the shape, initial precursor, and size of the nanoparticles. In Figure 3, the UV-Vis results are shown for selected optimum silver nanoparticle samples prepared using different extracts (i.e., BBE and PPE). The outcomes are elucidated after a set of optimization experiments (i.e., the liquid volume ratio, temperatures, serial extract dilutions, and reaction time). Figure 3a demonstrates the UV-Vis absorbance at the studied wavelengths (300–800 nm). The SPR of the Ag NPs synthesized with the employed extracts (i.e., BBE and PPE) displays a sharp peak located at ~420. The Ag-BBE sample possesses λ_max_ at ~419 nm, while in the case of the Ag-PPE sample, the value of λ_max_ is red-shifted to ~433 nm. The presence of the marked Ag SPR peak verifies the successful formation of the Ag NPs using BBE and PPE, which coincides with former research results [10,11,12,13,14,15]. The noticeable red shift in the SPR peak position may be attributed to the different phytochemical concentrations in the tested extracts that may have affected Ag NP formation. The wavelength shift gives possible variations in the size of the synthesized samples (Ag-BBE and Ag-PPE). The presence of a single peak occurrence reflects the spherical shape of the nanoparticles [35]. The successfully formed nanoparticles validate the suitability of the tested plant extracts (BBE and PPE) as capping and reducing agents for the desired green synthesis of the silver nanoparticles. The distinctive shape of the UV-Vis absorbance (i.e., symmetry, peak intensity, red/blue shift, FWHM, etc.) highlights the nature of the synthesized nanoparticles in several aspects [64]. It is worth mentioning that higher absorbance intensity means a higher absorbed wavelength and the easier excitation of electrons. As represented in Figure 3a, the Ag-BBE extract has a higher intensity than the Ag-PPE extract. This proves the progressive reaction of Ag^+^ reduction to Ag^0^, affording increased growth of nanoparticles [62]. The size of the nanoparticles is more closely related to the symmetry and FWHM (full width at half-maximum) of the peak. The higher symmetry of the peak branches indicates monodispersed particles with a uniform size distribution. In addition, FWHM governs the size and shape dispersity of the nanoparticles. The FWHM in the Ag-PPE sample has increased, which means the possible increase of NP size polydispersity [62,65]. The Ag-PPE samples are red-shifted, revealing the ripening of the nanoparticles, the possible formation of new bonds, and likely the elevated size of the nanoparticles [66,67]. Table 1 presents the key characteristics of the UV-Vis spectra of both Ag samples. To sum up, both extracts are applicable for the green synthesis of Ag NPs, while the BBE is likely to be more efficient in producing an increased number. The monodispersed distributed size of the bandgap can be extracted from the UV-Vis absorbance curve, commonly known as the Tauc plot [68,69,70]. The equation is mathematically expressed as in [70]: the silver nanoparticles exhibit a faster approach. For further insights into the optical specifications, Equation (1) was used to provide more information about the optical bandgaps of the green NPs.

(1) $$\left\{{\left(F\left(R_{\infty }\right)h\nu \right)}^{2}=\left[\beta \left(h\nu -E_{g}\right)\right]\right\}$$

The delivered parameters in this equation are *F*(*R*_∞_), *h*, *ν*, *β*, and *E_g_* which denote the Kubelka–Munk function, Planck’s constant (~6.62 × 10^−34^ Js), the frequency, energy-independent constant, and the optical bandgap. Note that:

(2) $$F\left(R_{\infty }\right)=\frac{k}{S}=\frac{{\left(1-R_{\infty }\right)}^{2}}{2R_{\infty }}$$

where *R*_∞_ represents the reflectance percentage. After completing the calculations, the plot was obtained, as depicted in Figure 3b. From the tangent of the linear section of the graph, we can determine the energy bandgap of both the Ag-BBE and Ag-PPE samples, as illustrated in Figure 3b, Table 1. The energy gap values are close to those in previous research [69]. Silver nanoparticles can modulate the viability of miscellaneous microorganisms and living cells [69]. Silver-mediated nanoparticles, with their bandgap modulation affinity, are convenient for photocatalytic, antimicrobial, degradation, and photoelectrochemical applications.

### 3.3. X-ray Diffraction (XRD) Analysis (Crystallographic Properties)

**Figure 4:**
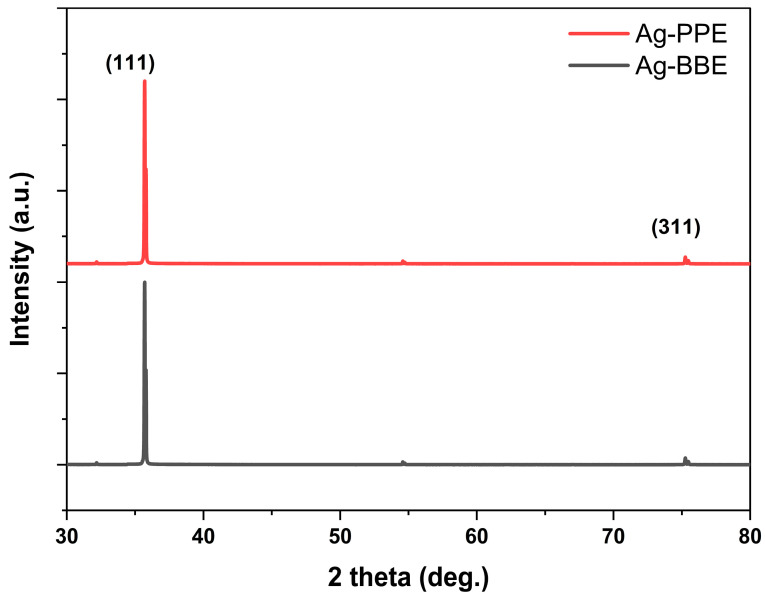
The full diffraction pattern of the green-synthesized silver nanoparticles (Ag-BBE: **down**), and (Ag-PPE: **up**).

XRD is an essential device for revealing the crystallographic structure, phase, and key lattice parameters of a structure through the obtained diffraction pattern. Many materials can be explored by XRD, such as metals, metal oxides, polymers, thin films, and nanocomposites [70,71]. Figure 4 elucidates the diffraction patterns of the synthesized silver nanoparticles in the Ag-BBE and Ag-PPE samples. At first glance, the crystallinity of the nanoparticles is demonstrated through the occurrence of high-intensity sharp peaks. Distinctive peaks with a slight shift of the silver nanoparticles in both samples are located at ~35.7° and 75.2°, which can be attributed to the diffraction planes of (111) and (311), in accordance with the silver #JCPDS card No. 04-0783 [72,73]. The dominance of the (111) plane affords an FCC structure of silver with a lattice constant of 0.408 nm [72,73]. The absence of two peaks is evident in the patterns (i.e., (200) ~44° and (220) ~64°), whereas less intense peaks at ~32.1°, and 54.5° are apparent. While the XRD results confirm the effectivity ability of the tested extracts in the green synthesis processing of Ag NPs, these reduced-intensity peaks might arise as a consequence of the extract, which involves the organic compounds responsible for Ag NP reduction and stabilization [61,72].

### 3.4. Transmission Electron Microscope (TEM): Morphological Properties

**Figure 5:**
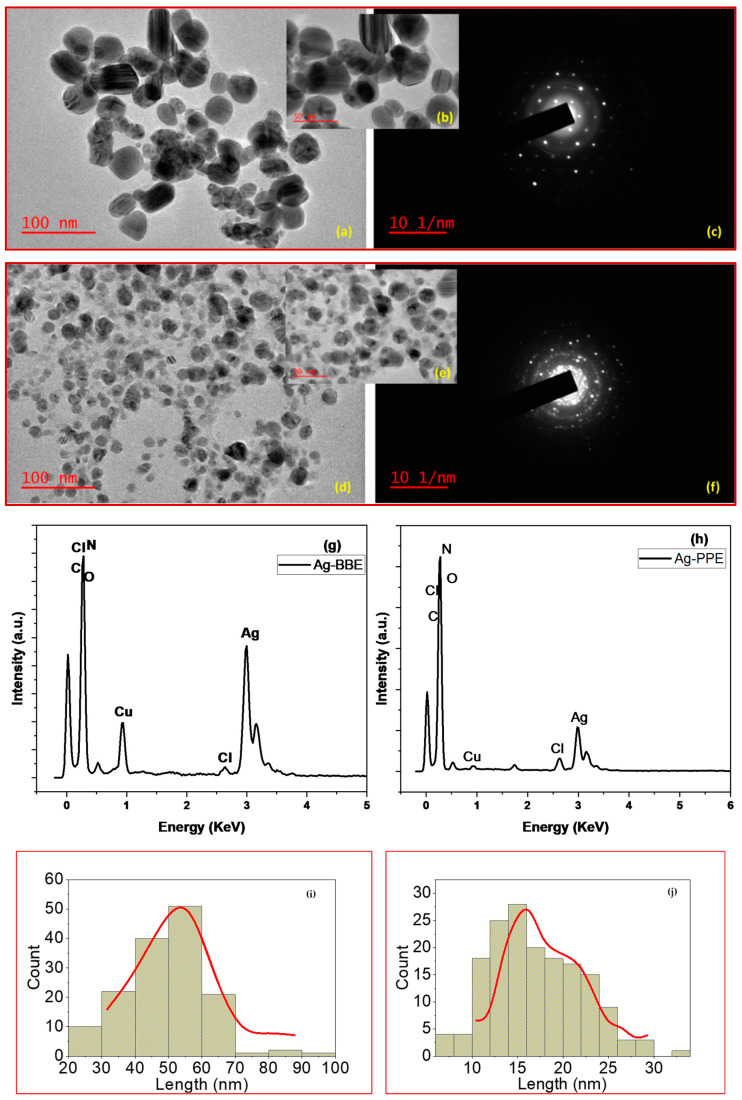
(**a**,**b**) The TEM of the Ag-BBE synthesized sample; (**d**,**e**) the TEM of the Ag-PPE sample; (**c**,**f**) the SAED of the Ag-BBE and Ag-PPE prepared silver nanoparticles, respectively; (**g**,**h**) the EDX spectra of the silver nanoparticles attained by BBE and PPE extracts, respectively; (**i**,**j**) the histograms of the particle size distribution for both the Ag-BBE and Ag-PPE nanoparticles, respectively.

The transmission electron microscope (TEM) is a familiar instrument that can illustrate the morphology and nature of the prepared nanomaterials, thin films, and nanocomposites. The shape and size of the produced silver nanoparticles in the Ag-BBE and Ag-PPE samples are depicted in Figure 5. As represented in this figure, their spherical shape and rounded particles, uniform distribution, and the absence of agglomeration are clear. However, the green-synthesized Ag-BBE sample, shown in Figure 5a–c, demonstrated particles greater in size than in the Ag-PPE sample, as shown in Figure 5d–f. The SAED represented in Figure 5c,f unveils the crystalline nature of the Ag-BBE and Ag-PPE samples, which is in agreement with the reported XRD results. Furthermore, the EDX detector in the TEM device authenticated the elemental composition of the synthesized green Ag NPs. The EDX represented in Figure 5g,h of both routes demonstrates the correlation between the acquired ratio of elements and the initial concentrations. The strong spectral signal of silver residing around 3 keV corresponds to the silver nanoparticles’ SPR absorption spectrum [69,70,72]. Notably, the intensity of the BBE-employed extract is greater than that of the PPE-employed extract, which reinforces the suitability of the BBE extract for a faster, more reliable, and cost-effective acquisition route. Other elements as Cu may be attributed to the residence of the carbon grid used during the TEM morphological examination. Lastly, the existing percentages of Cl, C, O, and N might emerge from the employed extracts in the course of preparation [74]. The C and N are commonly present in the biomolecules of the natural extracts, while the presence of O may either be attributed to the plant’s biomolecules, or may come from the aqua solution used during the extraction process. Further analysis via ImageJ 1.44 software was used to produce histograms showing the particle size distribution. The histograms for both Ag-BBE and Ag-PPE samples are shown in Figure 5i,j. The Ag-BBE particle size revealed a range of 20–70 nm, while the Ag-PPE prepared nanoparticles ranged from 10 to 30 nm. The Ag-BBE samples have an average particle size of 49.4 nm, while the latter Ag-PPE has a reduced particle size of 17.05 nm. Apparently, polydispersed size is evident in the Ag NPs-PPE synthesized sample, as shown in Figure 5d,e. Conversely, the monodispersed size of the nanoparticles is demonstrated for the Ag-BBE-formed nanoparticles in Figure 5a,b. These results are consistent with the expected outcome from the UV-Vis spectra. The morphological investigation revealed the potential ability of both tested extracts in the green synthesis of Ag NPs. It also revealed the effectivity of PPE and BBE as potential reducing and capping agents in the green synthesis process [75]. However, it was evident that the changes in the size and the shape of the synthesized Ag-BBE and Ag-PPE NPs could be disputed when using the same optimal synthesis conditions. This can be attributed to the difference in the tested extract’s composition and the extract’s biomolecules involved in the synthesis process. The reduced ability of PPE might have stronger abilities compared with the BBE, which may lead to faster nucleation and smaller nanoparticles. In addition, the capping agent potential of the PPE component can prevent the growth of nanoparticles beyond a certain size. More information about the extract’s composition will be discussed below, as it may facilitate understanding the mechanism of formation. In conclusion, the presented TEM results are in agreement with other reported studies, wherein the effect of the involved biomolecules affected the Ag particles’ size distribution [76]. The current results revealed the vital role of the tested extracts on the size, shape, crystallinity, degree, and orientation of the synthesized NPs. The results are in agreement with the critical vital effect of the used extracts on the characteristic properties, including the size and shape, of the synthesized Ag NPs [77].

### 3.5. Raman Spectroscopy (Vibrational Properties)

**Figure 6:**
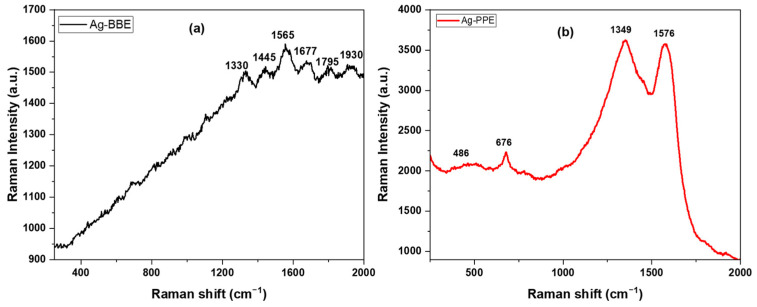
The Raman spectroscopy investigation of the green-prepared silver nanoparticles (**a**) Ag-BBE and (**b**) Ag-PPE nanoparticles.

Raman spectroscopy device investigation has become more familiar recently, owing to the current focus on applications, not instrumentation restrictions. Raman spectroscopy can explore the key specifications of versatile types of materials, encompassing powders, pellets, and emulsions. A characteristic property of Raman spectroscopy is the as-prepared investigation of the tested samples [78]. Inherently, Raman spectroscopy highlights chemical identification, the nature of the molecular structure, and bonding impacts [79]. Figure 6 represents the Raman spectroscopy patterns for the green-synthesized Ag-BBE and Ag-PPE NPs. The silver nanoparticles were produced using the extracts, which behaved as a surfactant for the synthesis of Ag NPs. As depicted in Figure 6b, the pattern of Ag-PPE NPs has four characteristic bands that are located at 486, 676, 1349, and 1576 cm^−1^. The represented results are in correlation with the standard reported Raman vibrational bands of green-synthesized Ag NPs [80]. The vibrational modes that emerged at 486,676 cm^−1^ can be attributed to the stretching vibration of C-N-C and C-S-C that was reported earlier [80,81]. The latter bands situated at 1349 and 1576 cm^−1^ are allocated to the C=O carboxylic group stretching vibration for symmetric and anti-symmetric vibrations, correspondingly [80,82]. In the same way, the presence of silver nanoparticles is reinforced by the aforementioned bands; however, a slight shift in locations might emerge as a consequence of interacting with the organic functional groups of the extract and initial Ag precursor [83,84]. Compared to the Ag-PPE NPs, the silver nanoparticles equipped by the BBE extract, Ag-BBE NPs, demonstrate much reduced Raman spectroscopy intensity, as demonstrated in Figure 6a. The Ag-BBE sample revealed characteristic bands that are located at 1330, 1445, 1565, 1677, 1795, and 1930 cm^−1^. The 1330 cm^−1^ may be attributed to the D-band associated with disordered carbon [85] or shifted C=O [80,82], while the band located at 1445 cm^−1^ belongs to the CH_2_ or CH_3_ bending vibrations of the organic biomolecules of the used extract [86], BBE. Both the bands located at 1565 and 1795 cm^−1^ may be ascribed to the C=C stretching vibration, while the band at 1677 cm^−1^ is correlated to the C=O stretching vibration band [87]. Lastly, the band at 1930 cm^−1^ may be due to residual nitrate from the synthesis process [88]. The Raman data are in good agreement with the reported elemental analysis revealed in the reported EDX data. The Raman data in Figure 6a revealed the great influence of the used extract (BBE) on the vibrational characteristic properties of the synthesized Ag NPs. The Ag-BBE and Ag-PPE samples were prepared under the same conditions (including the ratio, concentration, temperature, aging time, etc.). Hence, we believe that the variance in the Raman band intensity can be attributed to the differences in the extracts’ compositions. The active components and the active functional group in each extract play a vital role in the formation and characteristics of the NPs. This may occur through the reducing, nucleation, and capping steps.

Based on the represented results gained through different characterization techniques, both the tested extracts BBE and PPE reveal an ability regarding the formation of Ag NPs. Looking into the extract components may help to understand the possible mechanism of NP formation. The PPE contains different phenolic compounds (phenolic acids, flavonoids, and tannins); anthocyanins; polyphenols; pectins, carotenoids, and fiber. The composition of the PPE varies depending on several factors, such as the extraction process and cultivation [89]. It has been reported that the phenolic compounds possess hydroxyl groups (-OH) that can act as reducing agents. During interactions with silver ions (Ag+), they can donate electrons, causing the reduction of silver ions into silver nanoparticles (Ag NPs). Additionally, phenolic compounds may play a vital role as capping agents, preventing the nanoparticles from aggregating, and stabilizing them in solution [90]. Conversely, BBE possesses anthocyanins. Specifically, BB flowers contain four anthocyanins: cyanidin-3,5-*O*-diglucoside (cyanin), peonidin-3,5-*O*-diglucoside (peonin), cyanidin-3-*O*-glucoside, and cyanidin-coumaroylglucoside-pyruvic acid. The most abundant anthocyanin in the flowers is cyanidin-3,5-*O*-diglucoside, followed by peonidin-3,5-*O*-diglucoside, cyanidin-3-*O*-glucoside, and cyanidin-coumaroylglucoside-pyruvic acid. Anthocyanins are the component most likely to play a role in the synthesis of Ag NP formation. They have a reduced ability due to the presence of hydroxyl groups (-OH) in their structure. These hydroxyl groups can act as reducing agents, similar to how phenolics donate electrons to silver ions (Ag+) for the formation of Ag NPs.

### 3.6. Antimicrobial Activity and Statistical Analysis

The experiment was designed to measure and compare the effects of two green-synthesized silver nanoparticles (Ag-BBE and Ag-PPE NPs) on four selected microbes (*SA*, *CA*, *S. epi*, and *EF*). The measure of the effect was via the ZOI (mm) over uniformly cultured 100 µL microbes, adjusted to 0.5 Mcf on a Mueller–Hinton agar plate (15 × 90 mm). Initially, the Ag-BBE and Ag-PPE NPs were compared to their relative aqueous plant extracts and CHX to determine their activity. Thereafter, the two NPs were compared. For Ag-BBE, the ranges (mm) for the effect on the ZOI for Ag-BBE, BBE, and CHX were as follows:

*SA*: (12 ≤ ZOIAg-BBE ≤ 13), (16 ≤ ZOIBBE ≤ 18), and (22 ≤ ZOICHX ≤ 25)

*CA*: (9.5 ≤ ZOIAg-BBE ≤ 17), (19 ≤ ZOIBBE ≤ 23), and (12 ≤ ZOICHX ≤ 20)

*S. epi*: (16 ≤ ZOIAg-BBE ≤ 17), (20 ≤ ZOIBBE ≤ 24), and (22 ≤ ZOICHX ≤ 23)

*EF*: (12 ≤ ZOIAg-BBE ≤ 14), (10 ≤ ZOIBBE ≤ 12), and (20 ≤ ZOICHX ≤ 22)

These readings indicate that Ag-BBE had the lowest effect for SA, CA, and *S. epi*, while the effect was intermediate for EF. However, this does not relegate the activity of Ag-BBE. This is because NPs can be standardized and may not be prone to batch-to-batch variations, as seen in plant extracts. Also, NPs have been reported to discharge their activity at a steady rate [91,92]. Higher activity due to the plant extract may also emanate from a broad spectrum of bioactive compounds, which can have synergistic effects, while nanoparticles have a narrow standard spectrum [93].

For Ag-PPE, the ranges (mm) for the effect on the ZOI for Ag-PPE, PPE, and CHX were as follows:

*SA*: (11 ≤ ZOIAg-PPE ≤ 15), (22 ≤ ZOIPPE ≤ 26), and (23 ≤ ZOICHX ≤ 25)

*CA*: (11 ≤ ZOIAg-PPE ≤ 16), (22.5 ≤ ZOIPPE ≤ 29), and (19 ≤ ZOICHX ≤ 23)

*S. epi*: (17 ≤ ZOIAg-PPE ≤ 20), (22 ≤ ZOIPPE ≤ 25), and (22 ≤ ZOICHX ≤ 24)

*EF*: (11 ≤ ZOIAg-PPE ≤ 14), (ZOIPPE = 8), and (18 ≤ ZOICHX ≤ 21)

The average readings for Ag-PPE indicated the lowest activities on *CA*, *SA*, and *S. epi*; however, relatively high activity with *S. epi* was seen compared to the others. The activity on *EF* was intermediate between the other two treatments. Overall, the nanoparticles from both plants demonstrated lower levels of activity than their respective plant extracts. This may be expected because the two modalities may have different mechanisms of action, spectrums of activity, and general effectiveness. Plant extracts contain a diversity of bioactive compounds, such as alkaloids, flavonoids, and terpenoids, which may disrupt cell membranes, inhibit essential enzymes, or interfere with the genomic activities within microbial cells [94]. These compounds may have synergistic effects, enhancing antimicrobial activity. Some of these compounds may not be carried over into the nanoparticle format, thereby lowering their activity [95]. Further research needs to be undertaken to fully understand and optimize nanoparticle use as an antimicrobial agent. However, it has already been demonstrated that nanoparticles generally have an advantage over plant extracts, with prolonged antimicrobial activity and biofilm penetrative capacity. It has been reported that nanoparticles tend to accumulate in the cytoplasm, thus extending their period of activity [96].

However, the experiment aimed to compare the activity of the two NPs: Ag-BBE and Ag-PPE. The assumption was posited that there would be no differences in the effects due to the two treatments. The ANOVA revealed that there was an overall significant difference in the zones of inhibition between the two silver nanoparticle treatments on the four microbes at the *p* < 0.05 level for the eight groups F (2.08) = 57.48, *p* = 2.26 × 10^−36^. Hence, a post hoc test was performed to elucidate the interactions with significant differences, as well as those where there were none.

**Figure 7:**
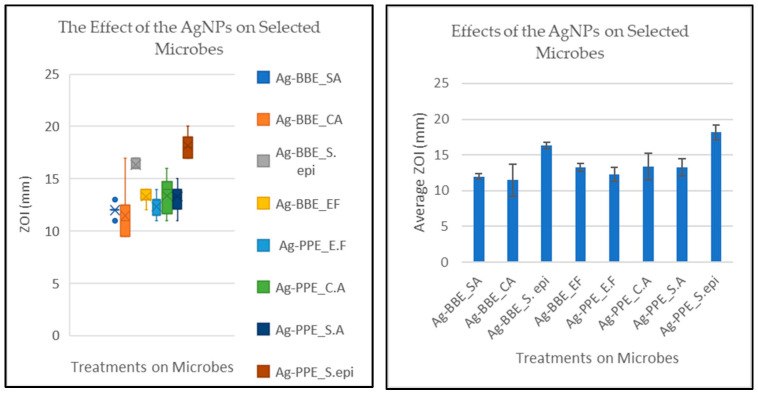
Box plot presenting the activities of Ag-BBE and Ag-PPE on *S. aureus* (*SA*), *C. albicans* (*CA*), *S. epidermidis* (*S. epi*), and *E. faecalis* (*EF*) as zones of inhibition (ZOI), together with column charts presenting the related averages.

Overall, the lowest effect was observed on *CA* treated with Ag-BBE and the highest effect was seen on *S. epi* treated with Ag-PPE (Figure 7). The Bonferroni test (adjusted α = 0.00625) further revealed significant differences between the two treatments, albeit on different microbes (mean difference = 6.68, 95% CI: 11.47–18.16, *p* = 1.57 × 10^−11^). Analysis of these interactions is important in elucidating the location of the differences, as indicated by the ANOVA.

Generally, post hoc analysis revealed significant differences in the effects of the treatments. A synopsis of the results is presented in Table 2 with reference to Ag-PPE_*SA*. The differences in the antimicrobial activity of nanoparticles across different microbes are well-documented in the literature. Differences depend on the microbe and the activity of the nanoparticles, among other factors [97,98]. An interaction without significant differences is reflected in Table 3 with Ag-PPE_*SA* and with Ag-BBE_*CA* in Table 2. The lack of differences suggests that these treatments may be equally effective, at least in vitro. This advocates equal possibilities for choosing either of the two treatments, given co-infections with the two relevant microbes. This parity of chance increases the possibilities of treatment while limiting the risk of antimicrobial resistance [99].

Although these nanoparticles demonstrated low activity compared to CHX, they may still be useful for controlling microbial growth during infections. Controlling microbial growth may be desirable in some cases instead of killing the microbes, thus leaving the immune system free to clear the infection, e.g., in cases of Gram-negative infections [100]. In other cases, it may not be desirable to eliminate the microbe at all, e.g., when the aetiological agent involved is normal flora. In these cases, such nanoparticles may be developed as precision antimicrobial treatments [101].

While the treatments in Table 3 had similar activities, it is worth noting that there were mean differences associated with each of these interactions, suggesting an edge of one treatment over the other where appropriate. The limitation in concluding these points from this study may lie in the lack of cytotoxicity testing of these two interventions. In addition, biocompatibility studies also need to be conducted to further characterize these nanoparticles.

Both treatments demonstrated the highest activity on *S. epidermidis.* However, the two treatments varied in terms of the lowest activities with *C*. *albicans* and *E. faecalis* for Ag-BBE and Ag-PPE NPs, respectively, as shown in Figure 7.

However, the focus of this study was comparing Ag-BBE and Ag-PPE. Thus, owing to these limitations, the effects of the two treatments are specifically reported in this article as a comparison with each microbe. Hence, the four comparisons are detailed below in Table 4. The Bonferroni post hoc analysis was read for a comparison of the effect of the two treatments on the same organism, e.g., the ZOI for Ag-BBE were compared to those for Ag-PPE. The test revealed significant differences across all four test organisms, as shown in Table 4.

These results indicate that Ag-BBE had a lesser effect across the tested microbes, except on *E. faecalis.* These discrepancies could be related to several factors, including the nature of the nanoparticles (e.g., size and surface charge) as well as the nature and physiology of the microbes used. This suggests that when the intervention is intended to treat polymicrobial infection, one would prefer to use Ag-PPE over Ag-BBE, while Ag-BBE may be preferred when specifically targeting *E. faecalis*, as in root canal treatment, where the organism is notorious for causing treatment failure and re-infection [10,102].

Given the limitations and the narrow perspective above, it may be concluded that Ag-PPE demonstrated superior antimicrobial activity over Ag-BBE. However, further antimicrobial assays with a more comprehensive list of microbes and cytotoxicity assays still need to be conducted. Furthermore, due to the antimicrobial promise that the two microbes possess, cytotoxicity and biocompatibility assays must be conducted on them.

## 4. Conclusions

In conclusion, the current research successfully revealed the effect of using two different extracts for the green sustainable synthesis of silver nanoparticles. *Callistemon citrinus* (bottlebrush) flower extract (BBE) and *Punica granatum* (pomegranate) peel extract (PPE) were selected as potential reducing and capping agents. Herein, the screening and optimization step identified the optimal synthesis conditions, and the influence of the extract’s concentration, volume ratio, and reaction time was investigated. The green synthesis process of Ag NPs was carried out under the same optimized conditions to reveal the effect of the plant type on the different NP properties. The synthesized Ag samples were investigated using various analysis techniques, such as UV-Vis and Raman spectroscopies, XRD, and TEM. Antimicrobial testing was utilized to assess the NPs’ properties. The selected characterization techniques evaluated the optical, vibrational, crystalline structure, and morphological properties of the synthesized silver nanoparticles (Ag-BBE, and Ag-PPE), using the two selected extracts (BBE and PPE). The UV-Vis data revealed the distinct SPR absorption peaks of the Ag NPs, reflecting peaks at 419 and 433 nm for the Ag-BBE and A-PPE samples, respectively. Furthermore, the UV data facilitated the calculation of the energy bandgaps, producing 2.64 eV (Ag-BBE) and 2.51 eV (Ag-PPE). The crystalline nature and phase formation of the synthesized Ag NPs were confirmed by XRD. The XRD results illustrated an fcc structure for both the Ag-BBE and Ag-PPE samples. Furthermore, a direct investigation of the effect of the extracts on the size and shape of the particles was demonstrated using TEM. The TEM results revealed non-agglomerated particles for both synthesized samples (i.e., BBE and PPE). This reveals the potential effect of BBE as well as PPE as a capping and reducing agent. However, there was a difference in the particle’s size and shape, depending on the extract used for the synthesis of Ag NPs. Additional results about the elemental composition, crystallinity confirmation, and size distributions were provided by the EDS data, SAED images, and the represented histograms, respectively. The vibrational properties were examined using Raman spectroscopy. This provided a clear overview of the interaction between the extract’s biomolecules and the silver NPs. There was an obvious effect of the extracts on the Raman peaks of the synthesized Ag NPs. The biological investigation of antimicrobial activity against four oral microbes, *Staphylococcus aureus* (*SA*), *Candida albicans* (*CA*), *Staphylococcus epidermidis* (*S. epi*), and *Enterococcus faecalis* (*EF*), was successfully reported. The ZOI assay, followed by further statistical analysis, were applied and revealed the superior antimicrobial activity of the Ag-PPE NPs over the Ag-BBE NPs. Further investigation and a deeper understanding of the potential effects of the plant’s biomolecules for the development of green nanoparticles with the desired properties are essential.

## Figures and Tables

**Table 1:** UV-Vis absorbance curve characteristics for both of the employed extracts used in silver nanoparticle preparation.

Silver NPs	λ_max_ (nm)	A at λ_max_	FWHM (nm)	FWHM Range (nm)	Energy Gap (eV)
Ag-BBE	419	2.24	at 1.595 = 61.5	333–496	2.64
Ag-PPE	433	1.94	at 1.264 = 99.3	331.4–523.5	2.51

**Table 2:** Comparison of the effects of Ag-BBE and Ag-PPE on each microbe, with Ag-BBE_SA as a reference.

Reference	Analysis	Bonferroni Post Hoc Test *t*-Test (Adjusted α = 0.00625)			
*S. aureus* (Ag-BBE) 12 mm	Treatment Interactions	Means (mm Ø)	t Crit.	P (T ≤ t)	Decision
*C. albicans*	Ag-BBE_*CA*	11.47	1.73	0.16699	No sig. diff.
*S. epidermidis*	Ag-BBE_*S. epi*	16.33	1.70	6.57 × 10^−25^	Sig. diff.
*E. faecalis*	Ag-BBE_*EF*	13.28	1.70	5.54 × 10^−9^	Sig. diff.
*E. faecalis*	Ag-PPE_*EF*	12.31	1.73	0.12192	No sig. diff.
*C. albicans*	Ag-PPE_*CA*	13.38	1.75	0.004399	Sig. diff.
*S. aureus*	Ag-PPE_*SA*	13.28	1.73	0.000264	Sig. diff.
*S. epidermidis*	Ag-PPE_*S. epi*	18.16	1.73	4.11 × 10^−15^	Sig. diff.

**Table 3:** Interactions in the activities of the Ag NPs that yielded no significant differences.

Reference Microbe	Analysis	Bonferroni Post Hoc Test *t*-Test (Adjusted α = 0.00625			
	Treatment Interactions	Means (mm Ø)	t Crit.	P (T ≤ t)	Decision
*C. albicans*	Ag-BBE_*CA*	11.47	1.71	0.07989	No sig. diff.
	Ag-PPE_*EF*	12.31			
*E. faecalis*	Ag-BBE_*EF*	12.31	1.71	0.025312	No sig. diff.
	Ag-PPE_*CA*	13.38			
*E. faecalis*	Ag-PPE_*EF*	12.31	1.70	0.008358	No sig. diff.
	Ag-PPE_*SA*	13.28			
*C. albicans*	Ag-PPE_*CA*	13.38	1.71	0.431637	No sig. diff.
	Ag-PPE_*SA*	13.28			

Note: Interactions displayed in this table indicate accepting the null hypothesis, i.e., that there is no difference between the activities of the two Ag NPs. These interactions exclude those presented in this table.

**Table 4:** Comparison of the effects of each of the two synthesized Ag NP samples within each microbe.

Microbe	Analysis	Bonferroni Post Hoc Test *t*-Test (Adjusted α = 0.00625			
	Treatment Interactions	Means (mm Ø)	t Crit.	P (T ≤ t)	Decision
*S. aureus*	Ag-BBE_*SA*	12	1.73	0.000264	Sig. diff.
	Ag-PPE_*SA*	13.28			
*C. albicans*	Ag-BBE_*CA*	11.47	1.69	0.00492	Sig. diff.
	Ag-PPE_*CA*	13.38			
*S. epidermidis*	Ag-BBE_*S. epi*	16.33	1.72	8.36 × 10^−7^	Sig. diff.
	Ag-PPE_*S. epi*	18.16			
*E. faecalis*	Ag-BBE_*EF*	13.28	1.71	0.001054	Sig. diff.
	Ag-PPE_*EF*	12.31			

## Data Availability

The data presented in this study are available on request from the corresponding author.
